# From data to decision: an interpretable machine learning model for optimizing RAI therapy in Graves’ hyperthyroidism

**DOI:** 10.3389/fendo.2025.1711029

**Published:** 2026-01-26

**Authors:** Lu Lu, Xiaojuan Wei, Yan Chen, Dongyun Meng, Shaozhou Mo, Zeyong Sun, Fengyang Song, Kehua Liao, Wentan Huang

**Affiliations:** Department of Nuclear Medicine, People’s Hospital of Guangxi Zhuang Autonomous Region, Nanning, Guangxi Zhuang Autonomous Region, China

**Keywords:** explainable AI, Graves’ disease, machine learning, precision medicine, radioiodine therapy, treatment outcome prediction

## Abstract

**Objective:**

Radioactive iodine (RAI) therapy is a cornerstone treatment for Graves’ hyperthyroidism (GH), yet failure rates remain significant due to the complexity of individual patient responses. Traditional fixed-dose or simple calculated-dose methods often fail to account for non-linear interactions among clinical features.

**Methods:**

We retrospectively analyzed data from 1,292 GH patients who received initial RAI therapy between June 2018 and July 2024. Comprehensive pre-treatment clinical, laboratory, and imaging data, including age, gender, FT4, 3-hour radioactive iodine uptake (RAIU 3h), thyroid weight, and thyroid receptor antibodies (TRAb), were collected. Stepwise regression with the Akaike Information Criterion (AIC) was employed for feature selection, identifying nine optimal predictors. Six machine learning algorithms were compared, with performance evaluated using AUC, Brier score, and Decision Curve Analysis (DCA). SHapley Additive exPlanations (SHAP) analysis provided model interpretability.

**Results:**

The final cohort, comprising 1,292 patients (61.3% female, median age 37 years), achieved a 75.8% remission rate. Nine significant variables were identified as optimal predictors: gender, age, history of antithyroid drug use, disease course over 2 years, total iodine dose (TID), free thyroxine (FT4), RAIU 3h, thyroid weight, and TRAb. Among the algorithms tested, the Random Forest (RF) model demonstrated superior performance, achieving an AUC of 0.950 on the independent test set and a Brier score of 0.067, indicating excellent discrimination and calibration. SHAP analysis confirmed RAIU 3h, FT4, age, and thyroid weight as the most influential features, providing clinical transparency.

**Conclusion:**

The developed interpretable machine learning framework offers a precise, personalized tool for predicting RAI outcomes, potentially guiding optimizing dosing strategies to reduce treatment failure.

## 1 Introduction

Graves’ disease (GD) is a common autoimmune thyroid disorder primarily causing hyperthyroidism, known as GD hyperthyroidism (GH) (1). This condition results from antibodies stimulating thyroid-stimulating hormone (TSH) receptors, leading to excess thyroid hormone production (2). GH is the most prevalent form of hyperthyroidism, characterized by increased nervous, circulatory, digestive, and metabolic activity (3). In China, its incidence ranges from 1.1% to 1.6% (4). Currently, hyperthyroidism is primarily managed through three clinical approaches: antithyroid drugs (ATD), radioactive iodine therapy (RAI), and surgical thyroidectomy. Given the invasive nature of thyroidectomy, it is associated with potential postoperative complications, including hypothyroidism, recurrent laryngeal nerve injury, and hypocalcemia (5), which limits its use. RAI is a widely accepted, non-invasive treatment in Western countries like the United States due to its high cure rates, low recurrence, short treatment duration, simplicity, safety, minimal side effects, and low cost (6). It is often the first choice or a key alternative for GH patients, especially those with adverse reactions to ATD, poor drug efficacy, frequent relapses, long disease duration, surgical contraindications or risks, liver damage, leukopenia, thrombocytopenia, atrial fibrillation, or periodic skeletal muscle paralysis (5).

RAI for GH has been used for over 70 years, but determining the optimal thyroid absorption dose remains a challenge. Traditional dosing often involves a fixed range of 185–555 MBq (5–15 mCi), which overlooks individual patient differences, potentially leading to suboptimal outcomes or side effects. The dosing formula used is [Z × thyroid size (g) × 100]/24-hour iodine uptake rate (RAIU), where Z is the planned Bq or μCi per gram of thyroid tissue, ranging from 3.7 to 7.4 MBq (100-200 μCi) (7). Accurate thyroid size measurement is essential, but there is debate over whether current methods, like microdose RAIU, accurately reflect high-dose I-131 dynamics and gland radiation sensitivity (5, 8). Clinically, the calculated dosage results are often considered in conjunction with the patient’s disease condition to determine the final dosage. For example, in cases of prolonged illness, a hard thyroid gland, or patients who have not recovered after initial treatment, an appropriate increase in dosage may be warranted, while for patients with a short illness duration or those who have relapsed after surgery, a reduction in dosage may be appropriate.

I-131 dosage for GH treatment is determined using either empirical fixed-dose methods or formula-based calculations. The fixed-dose approach applies the same dosage to all patients, ignoring thyroid size and iodine uptake, and lacks scientific validity (9). In contrast, formula-based methods consider these factors, offering more scientific grounding. However, they rely on accurate thyroid size and weight data, which current methods like radionuclide imaging, ultrasound, and palpation fail to provide precisely (10). Measurement uncertainty leads to inconsistent treatment outcomes, underscoring the need for further research. Personalized I-131 dosing for GH treatment is essential due to individual differences in absorption and metabolism. Customizing doses based on factors like thyroid size and iodine uptake improves effectiveness, minimizes side effects, and reduces radiation exposure. This approach prevents I-131 overuse, cutting drug waste, costs, and secondary treatments. It also shortens hospital stays and treatment cycles, easing demand on protective wards and improving resource efficiency, ultimately allowing more patients to be served. Adopting personalized I-131 dosing for GH improves treatment outcomes, safety, and cost-efficiency, while advancing precision medicine and meeting societal demands for quality healthcare. This approach promises broader future applications and can help address the shortage of protective wards in China’s nuclear medicine sector, offering both economic and social benefits.

The rapid advancement of computational technology has markedly contributed to the expanding field of research dedicated to the application of machine learning (ML) algorithms in the analysis of medical data (11–13). ML enables the processing of large-scale medical datasets, facilitating more precise analyses that enhance clinical decision-making (11). Recent studies consistently highlight the significant advantages of ML in disease prediction, diagnosis, and treatment evaluation (14–16). In a notable development, Moon et al. introduced a ML classifier named OncoNPC, which leverages multi-center targeted sequencing data from 36,445 known primary cancer samples to predict the primary cancer type in cases of cancer of unknown primary (CUP). OncoNPC initially validated the existence of shared genetic and prognostic features between CUP and known cancer types. Its classification capabilities hold the potential to inform and guide clinical decision-making processes (17). Attia et al. have developed an expedited ML methodology for the detection of atrial fibrillation in patients during sinus rhythm, employing standard 10-second 12-lead electrocardiograms. This model exhibited enhanced efficacy in identifying potential atrial fibrillation in patients with cryptogenic stroke (ESUS), outperforming traditional screening techniques such as B-type natriuretic peptide levels and the CHA2DS2-VASc score. As a result, this advancement provides an innovative, cost-effective, and non-invasive tool for atrial fibrillation screening and the management of patients with ESUS (18). While ML has shown promise in medical prognosis, few studies have compared advanced ensemble methods with classical statistical models in predicting RAI outcomes. This study aims to fill this gap by developing an interpretable ML framework, validating it against classical approaches, and identifying key predictors for non-remission.

## 2 Patients and methods

### 2.1 Study subjects

This study encompassed a cohort of 1711 patients who received treatment for GH at the Nuclear Medicine Department of the People’s Hospital of Guangxi Zhuang Autonomous Region between June 2018 and July 2024. The diagnosis of hyperthyroidism was established in accordance with the 2016 American Thyroid Association Guidelines for the Diagnosis and Management of Hyperthyroidism and Other Causes of Thyrotoxicosis (5). All participants underwent their initial administration of RAI therapy. The inclusion criteria were defined as follows: (1) a confirmed diagnosis of GH; (2) discontinuation of ATD for at least five days; (3) initial administration of RAI; (4) commitment to consistent follow-up for one year post-treatment; and (5) availability of comprehensive diagnostic and treatment records. The exclusion criteria encompassed: (1) individuals with thyroid weights exceeding 80 grams, as determined by a ^99m^TcO_4_^-^ thyroid SPECT scan, to ensure homogeneity within the study population; this is due to the fact that large goiters often necessitate surgical intervention or unique dosimetric protocols, which could introduce selection bias; (2) pregnant or lactating women; (3) individuals with a history of thyroid surgery; (4) patients unable to adhere to regular follow-up schedules; (5) patients diagnosed with granulocyte deficiency and/or liver failure; and (6) individuals with a history of malignancies.

### 2.2 Assessment of therapeutic efficacy

An initial assessment of therapeutic efficacy was conducted for all patients 4 to 8 weeks following RAI therapy. Subsequently, thyroid function was assessed every 4 to 8 weeks for a period of up to twelve months, or until the patient developed hypothyroidism and attained a stable condition following thyroid hormone replacement therapy. The effectiveness of RAI treatment was classified based on follow-up outcomes as follows: (1) Complete remission or clinical cure: Follow-up extending beyond six months with full resolution of hyperthyroid symptoms and normalization of serum free thyroxine (FT4) levels; (2) Hypothyroidism: Presence of hypothyroid symptoms and signs, with serum FT4 levels below the normal range and elevated thyroid-stimulating hormone (TSH) levels; (3) Partial remission: Reduction in hyperthyroid symptoms, partial resolution of signs, and decreased serum FT4 levels without normalization; (4) Ineffective: Ineffective responses was defined by either no significant improvement or a worsening of hyperthyroidism symptoms and signs, with no reduction in serum FT3 and FT4 concentrations. Outcomes of complete remission or clinical cure and hypothyroidism were classified as “Remission” (remission group), whereas partial remission and ineffective responses were categorized as “Non-Remission” (non-remission group).

### 2.3 Data collection

Demographic variables:

Age: Recorded in years at the time of initial ^131^I therapy.

Gender: Documented as male or female, with a code of “1” representing male and a code of “2” denoting female.

Clinical parameters:

(1) Thyroid hormones and TPOAb: These were measured using the UniCel DxI 800 Access Immunoassay System with a chemiluminescence method: TSH: 0.56-5.91 μIU/mL; T3: 0.92-5.91 nmol/L; T4: 69.71-163.95 nmol/L; FT3: 3.53-7.37 pmol/L; FT4: 7.98-16.02 pmol/L; TPOAb:<9.0 IU/mL.
(2) TRAb: Measured using the UniCel DxI 800 Access Immunoassay System, with a reference range of 0-1.75 IU/L.
(3) Evaluation of RAIU: This study evaluated thyroid iodine uptake rates using I-131, provided by Nanning Atomic High-throughput Isotope Co., Ltd. Prior to the evaluation, patients were instructed to refrain from consuming iodine-containing foods and medications for a period of 2 to 4 weeks. On the day of the assessment, patients ingested sodium I-131, with doses ranging from 2 to 10 μCi, while in a fasting state in the morning. Following ingestion, patients continued fasting for an additional 2 hours. Radioactivity measurements of the thyroid region were subsequently conducted at 3 hours and 24 hours post-administration using the NM-6110 thyroid function measuring instrument. The effective half-life (Teff) was determined from the sequential I-131 uptake measurements. Teff is defined as the time required for the I-131 activity within the thyroid gland to decrease to 50% of its initial value, accounting for the combined effects of physical decay and biological clearance.
(5) Thyroid weight: After intravenous injection of ^99m^TcO4^-^ (2-5mCi), thyroid imaging was performed 15–20 minutes later. The patient was positioned supine with a pillow under the shoulder and neck to hyperextend the neck and fully expose the thyroid. Images were collected using the Discovery NM/CT 670, equipped with a low-energy general collimator, a matrix size of 256×256, an energy peak of 140keV, a window width of ±10%, and a collection count of 300k. The region of interest (ROI) was delineated in the blue-purple interface of the thyroid color image using Xeleris post-processing software to obtain the thyroid area, height, and weight.

Treatment-Related factors:

History of ATD Therapy: The variable “ATD” represents the history of ATD usage, where a code of “0” signifies no prior use and a code of “1” indicates a positive history of use.

Administered ^131^I Dosage: This refers to the prescribed dose of ^131^I in millicuries (mCi), as well as the iodine dose per gram of thyroid tissue (IDPG) measured in megabecquerels per gram (MBq/g). The variable “IDPG” categorizes the iodine dose per gram of thyroid tissue, with a code of “1” denoting small doses (70-90 μCi/g) and a code of “2” indicating large doses (91-120 μCi/g).

Course of Disease: This refers to the duration of GH prior to ^131^I therapy. The variable “Disease_course” is defined by the length of the illness, with a code of “0” indicating a duration of two years or less, and a code of “1” denoting a duration exceeding two years.

### 2.4 RAI treatment dose

The procedure and its associated precautions were comprehensively communicated to all patients, with particular emphasis placed on the necessity of adhering to a low-iodine diet and avoiding medications containing iodide for a duration of 7 to 14 days prior to treatment. Furthermore, ATD were required to be discontinued at least one week before the administration of ^131^I therapy.

Our hospital employs a calculated dosage method to determine the I-131 treatment dose, administered using a fully automated ^131^I dispensing machine, based on the formula:


$$\text{I}-131\text{ treatment dose }\left(\mu \text{Ci}\right)=\;\frac{\text{Dose per gram of thyroid tissue }\left(\text{uCi}\right)\times \text{ Thyroid weight }\left(\text{g}\right)}{24\text{h thyroid uptake rate of I}-131\;\left(\%\right)}$$

According to their clinical condition, three expert nuclear medicine physicians prescribed the IDPG for each patient, generally between 70-120 μCi/g.

### 2.5 Feature selection

To ensure data quality, we utilized the MissForest algorithm to impute minor missing values (missing rate< 5%). This non-parametric imputation method, based on RF, facilitates the estimation of missing values in mixed-type data (19). Continuous variables were retained in their original form to preserve information granularity, addressing limitations associated with categorical conversion. To ascertain the most salient predictive factors and mitigate data dimensionality, we utilized a stepwise regression analysis employing a bidirectional elimination strategy, integrating both forward selection and backward elimination techniques. This method iteratively refines the model by incorporating variables that substantially enhance model fit while discarding those deemed statistically insignificant or redundant. The selection process was directed by the minimization of the Akaike Information Criterion (AIC), which optimizes the trade-off between model fit and complexity. The algorithm commenced with an initial model and persisted in the iterative procedure until the AIC score attained its minimum, indicating no further potential for improvement.

### 2.6 Model development, evaluation, and interpretation

In this study, we investigated six distinct ML algorithms to ascertain the optimal classifier for the dataset: XGBoost Classifier (XGB), Logistic Regression (LR), LightGBM Classifier (LGBM), Random Forest Classifier (RF), AdaBoost Classifier, and Decision Tree Classifier (DT). To enhance model performance and mitigate overfitting, a 5-fold cross-validation approach was implemented during the training phase. Hyperparameter optimization was performed using GridSearchCV to identify the optimal parameter configurations for each algorithm. The primary metric for model evaluation was the Area Under the Receiver Operating Characteristic (ROC) Curve (AUC). Additional evaluation metrics included accuracy, sensitivity, specificity, positive predictive value (PPV), negative predictive value (NPV), F1-score, and Cohen’s Kappa. The optimal threshold value was determined through ROC curve analysis. The DeLong test was utilized to statistically compare the AUCs across different models.

**Figure 1:**
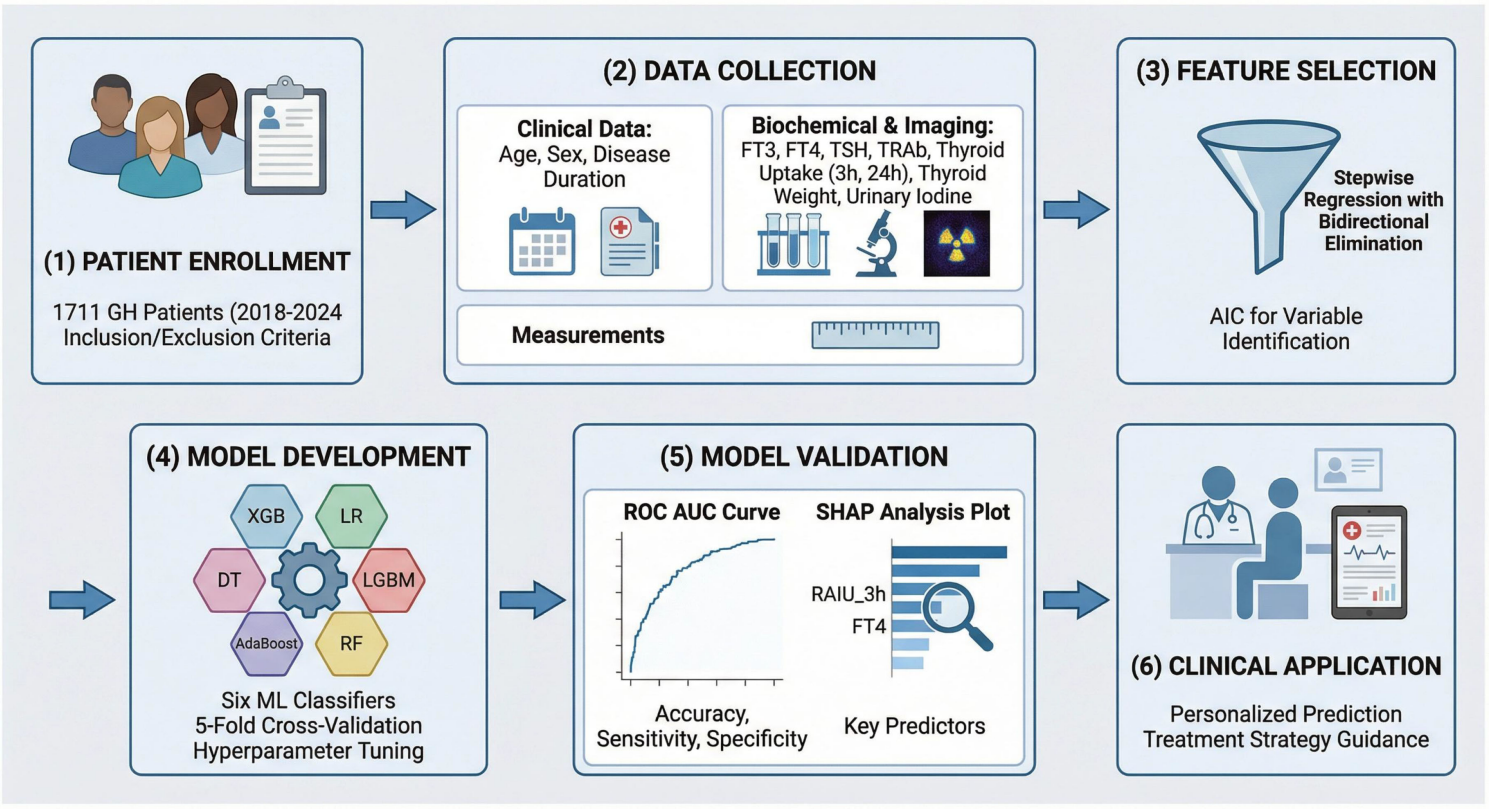
Study overview.

We constructed a SHAP (SHapley Additive exPlanations) summary plot, also known as a beeswarm plot, to illustrate the overall significance and directional impact of the features. In this visualization, features are ordered according to the sum of absolute SHAP values across all samples. Each point represents an individual sample, with color coding indicating the feature value (red for high values, blue for low values), and the position along the x-axis reflecting the feature’s impact on the model’s output (where positive SHAP values suggest an increased probability of the positive class, i.e., Label=1). Additionally, SHAP dependence plots were employed to assess the marginal effects of specific features, such as FT4 and TRAB, on the predicted outcomes, facilitating the identification of potential non-linear relationships and interaction effects among variables. To further illustrate the model’s clinical applicability in individual cases, we utilized SHAP force plots, or waterfall plots, to analyze specific samples from the test set. These plots decompose the prediction for an individual patient, demonstrating how each feature contributes to either elevating (positive force) or reducing (negative force) the prediction relative to the baseline (**Figure 1**).

### 2.7 Statistical analysis

Statistical analyses and data processing were executed using R software (version 4.2.3) and Python (version 3.11.4). Continuous variables were first assessed for normality; those following a normal distribution were expressed as mean ± standard deviation (SD) and compared using the independent samples t-test. Conversely, non-normally distributed data were presented as the median with interquartile range [M (P25, P75)], with group comparisons conducted via the Mann-Whitney U test. Categorical data were summarized as frequencies and percentages [n (%)], and differences between groups were analyzed using the Chi-square test or Fisher’s exact test. All statistical tests were two-sided, and a P-value of less than 0.05 was considered statistically significant.

## 3 Result

### 3.1 Patient characteristics

**Figure 2:**
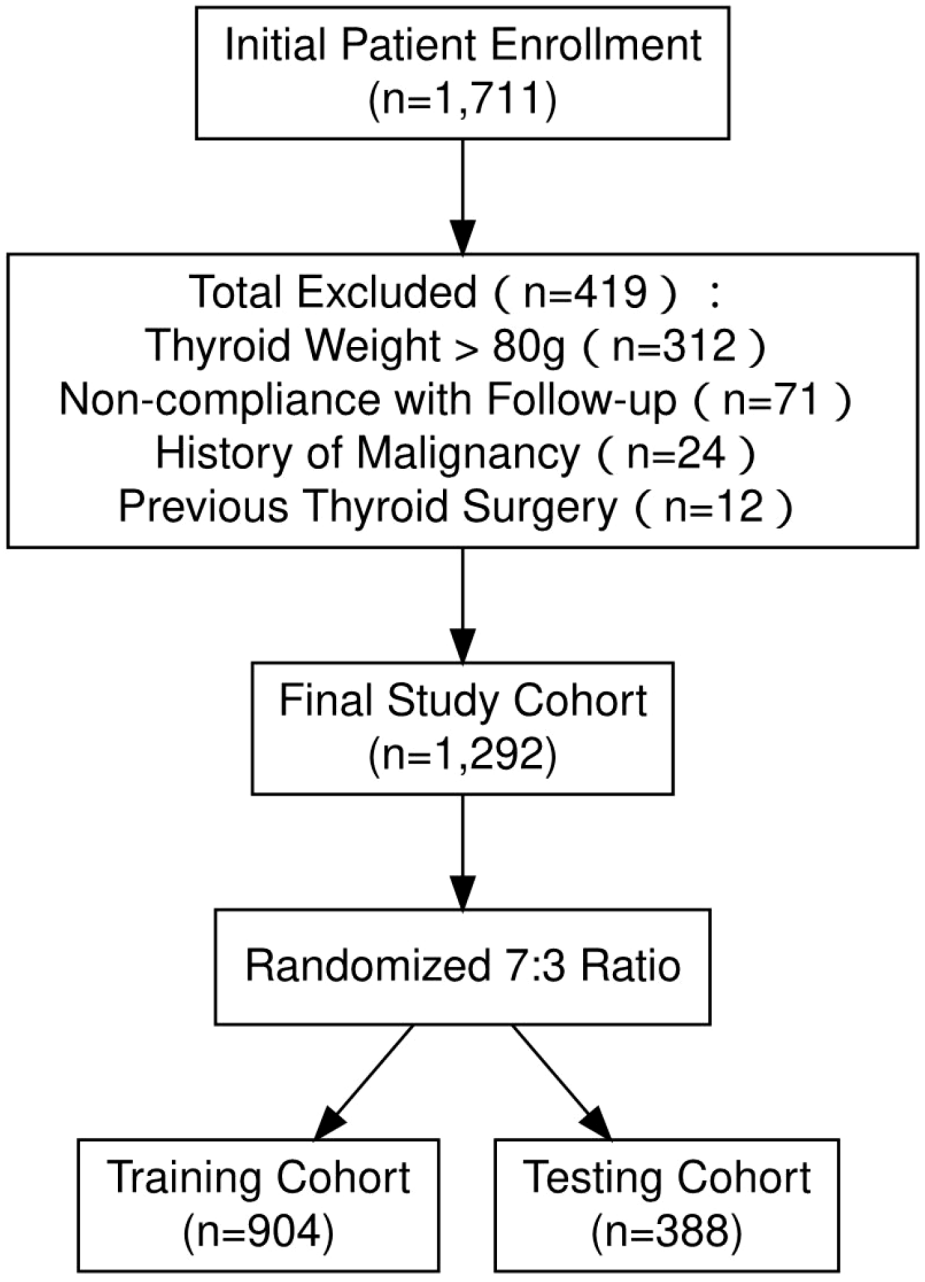
Patients enrollment flowchart.

In this study, an initial cohort of 1,711 patients was enrolled. However, 419 patients were subsequently excluded due to various factors: thyroid weight exceeding 80 grams (312 patients), non-compliance with follow-up protocols (71 patients), a history of malignancies (24 patients), and prior thyroid surgery (12 patients). Consequently, the final cohort consisted of 1,292 patients, who were stratified into a training cohort and a testing cohort in a 7:3 ratio (**Figure 2**). Within the training cohort, the median age was 37.00 years (interquartile range [IQR]: 29.00-43.00), with 369 males (40.82%) and 535 females (59.18%). Prior to receiving RAI, the mean FT3 level was 22.09 pmol/L (IQR: 15.02-30.69), while the median FT4 level was 53.95 pmol/L (IQR: 40.82-63.69). In the testing cohort, the median age was 37.00 years (IQR: 30.00-43.00), comprising 150 males (38.66%) and 238 females (61.34%). The FT3 level prior to RAI was 21.75 pmol/L (IQR: 14.24-30.13), and the median FT4 level was 53.19 pmol/L (IQR: 38.27-62.02). In the training cohort, a cure was achieved by 75.77% of patients (685 out of 904), while 24.23% (219 out of 904) did not attain a cure. In the training cohort, 75.77% of patients (685 out of 904) achieved a cure, whereas 24.23% (219 out of 904) did not attain this outcome. Similarly, in the testing cohort, 74.74% of patients (290 out of 388) were cured, while 25.26% (98 out of 388) were not. Further information is available in **Table 1**.

**Table 1:** Clinical baseline characteristics of the training set and testing set.

Characteristic	All (n=1292)	Training set(n=904)	Testing set (n=388)	P.overall
Age, Median [Q1-Q3]	37.00 [29.00;43.00]	37.00 [29.00;43.00]	37.00 [30.00;43.00]	0.772
TID, Median [Q1-Q3]	5.10 [4.41;6.00]	5.16 [4.44;6.00]	5.00 [4.36;5.71]	0.122
FT3, Median [Q1-Q3]	21.94 [14.71;30.57]	22.09 [15.02;30.69]	21.75 [14.24;30.13]	0.333
RAIU3h, Median [Q1-Q3]	68.94 [53.63;81.90]	69.25 [53.75;81.71]	68.26 [53.57;82.43]	0.882
RAIU24h, Median [Q1-Q3]	85.41 [76.63;92.80]	85.48 [76.85;92.73]	84.88 [75.50;93.08]	0.739
Thyroid_weight, Median [Q1-Q3]	48.00 [39.54;58.72]	48.30 [39.54;59.22]	47.02 [39.58;57.23]	0.335
Unrine_iodine, Median [Q1-Q3]	62.00 [58.99;68.97]	62.13 [59.00;68.97]	61.78 [58.74;68.88]	0.563
TPOAB, Median [Q1-Q3]	342.57 [69.08;690.36]	343.15 [63.84;710.37]	323.45 [71.53;665.66]	0.895
TRAB, Median [Q1-Q3]	16.77 [11.35;23.11]	16.76 [11.32;22.72]	16.87 [11.36;24.07]	0.693
the history of ATD usage, N (%):				0.658
No	496 (38.39%)	343 (37.94%)	153 (39.43%)	
Yes	796 (61.61%)	561 (62.06%)	235 (60.57%)	
Disease coursed>2 years, N (%):				0.849
No	1157 (89.55%)	811 (89.71%)	346 (89.18%)	
Yes	135 (10.45%)	93 (10.29%)	42 (10.82%)	
IDPG, N (%):				0.725
Small doses (70–90 uCi/g)	893 (69.12%)	628 (69.47%)	265 (68.30%)	
Large doses (91–120 uCi/g)	399 (30.88%)	276 (30.53%)	123 (31.70%)	
Gender, N (%):				0.507
Male	519 (40.17%)	369 (40.82%)	150 (38.66%)	
Female	773 (59.83%)	535 (59.18%)	238 (61.34%)	
Efficacy, N (%):				0.745
Remission	975 (75.46%)	685 (75.77%)	290 (74.74%)	
Non-Remission	317 (24.54%)	219 (24.23%)	98 (25.26%)	

### 3.2 Feature selection and multivariate analysis

Based on the stepwise regression analysis using the AIC criterion, nine significant variables were identified as the optimal predictor subset from the initial candidate features. These selected features included gender, age, history of ATD usage, disease course over 2 years, TID, FT4, RAIU 3h, thyroid weight, and TRAB.

The multivariate logistic regression results for these selected features are detailed in **Table 2**. The analysis revealed significant associations between these clinical parameters and the outcome. Specifically, disease course over 2 years (OR = 2.315, 95% CI: 1.401 - 3.823, P = 0.001) and history of ATD usage (OR = 2.187, 95% CI: 1.415 - 3.382, P<0.001) were identified as strong independent risk factors. Additionally, Age, TID, RAIU 3h, thyroid weight, and TRAB were positively associated with the outcome (all OR > 1). Conversely, gender (OR = 0.523, 95% CI: 0.349 - 0.785, P = 0.002) and FT4 (OR = 0.971, 95% CI: 0.959 - 0.983, P<0.001) demonstrated a negative association, serving as protective factors or negative predictors in the model. These nine features were subsequently utilized as the input variables for the development of ML models.

**Table 2:** Stepwise regression using forward and backward methods.

Variables	Partial regression coefficient	Standard error	z-value	OR(95%CI)	P value
(Intercept)	-10.871	2.641	4.116	0.000(0.000~0.003)	0.000
Gender					
Female				Reference	
Male	-0.648	0.207	3.133	0.523(0.349~0.785)	0.002
Age	0.043	0.009	4.882	1.044(1.026~1.062)	0.000
The history of ATD usage					
No				Reference	
Yes	0.783	0.222	3.520	2.187(1.415~3.382)	0.000
Disease coursed>2 years					
No				Reference	
Yes	0.839	0.256	3.277	2.315(1.401~3.823)	0.001
TID	0.239	0.116	2.064	1.270(1.012~1.593)	0.039
FT4	-0.029	0.006	4.595	0.971(0.959~0.983)	0.000
RAIU 3h	0.032	0.012	2.663	1.033(1.009~1.057)	0.008
Teff	0.491	0.299	1.642	1.635(0.909~2.939)	0.101
Thyroid weight	0.040	0.012	3.258	1.041(1.016~1.067)	0.001
TRAB	0.046	0.011	4.261	1.047(1.025~1.070)	0.000

### 3.3 RF: The best comprehensive discrimination and calibration capabilities

**Figure 3:**
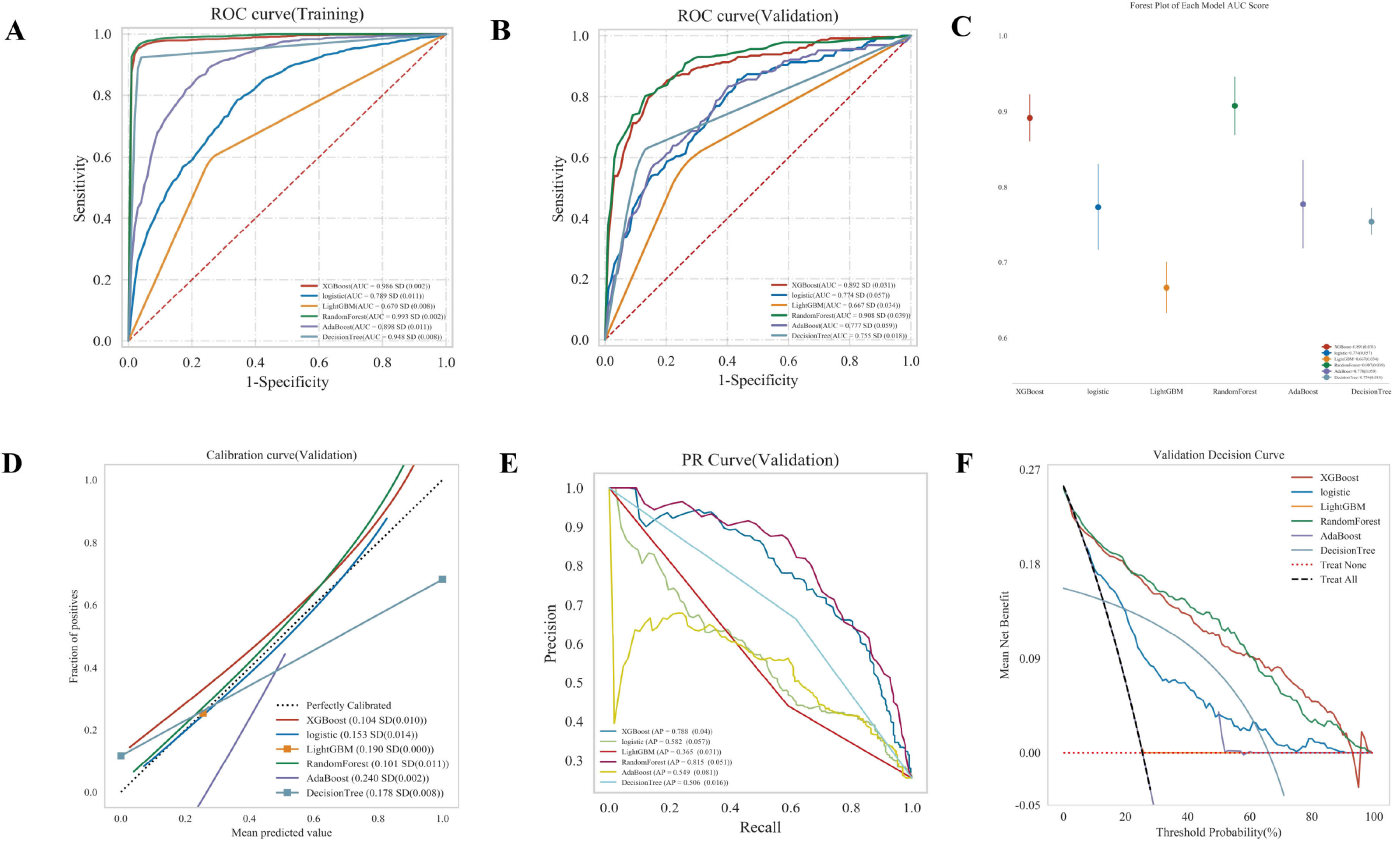
Comprehensive assessment of predictive performance, calibration, and clinical utility. **(A, B)** ROC analysis demonstrates the discriminatory ability of the models. The Random Forest model achieved the highest AUC in both the validation **(A)** and training **(B)** sets. **(C)** Comparison of AUC distributions among the six models. **(D)** Calibration plots showing the agreement between predicted risks and actual outcomes. Models closer to the diagonal line indicate better calibration. **(E)** Decision curves showing the net benefit of using the models across a range of threshold probabilities compared to default strategies. **(F)** Precision-Recall curves evaluating model performance, particularly focusing on the trade-off between precision and recall.

To identify the optimal ML algorithm for our predictive task, we conducted a comprehensive performance comparison among six models: XGB, LR, LGBM, RF, AdaBoost, and DT (refer to **Supplementary Tables S1, S2**). As illustrated in **Figure 3**, the RF model demonstrated superior performance across multiple evaluation metrics in the validation cohort.

In terms of discrimination ability, the RF model achieved the highest AUC. As shown in the validation ROC curves (**Figure 3A**), the RF model attained an AUC of 0.908 (SD = 0.039), outperforming the second-best model, XGB (AUC = 0.892, SD = 0.031), and significantly surpassing the other algorithms such as AdaBoost (AUC = 0.777) and DT (AUC = 0.755). The training set results (**Figure 3B**) further confirmed the robust learning capacity of the RF model, with an AUC of 0.993. The forest plot of AUC scores (**Figure 3C**) visually summarizes these findings, highlighting the RF model’s leading position with the highest mean AUC and stable confidence intervals.

Furthermore, the Precision-Recall (PR) curve (**Figure 3E**), which is particularly informative for evaluating classifier performance, showed that the RF model yielded the highest Average Precision (AP) of 0.815 (SD = 0.051). This indicates that the RF model maintained high precision even at varying levels of recall, superior to XGB (AP = 0.788) and LR (AP = 0.582).

Model calibration was assessed to evaluate the agreement between predicted probabilities and observed outcomes. The calibration curve (**Figure 3D**) revealed that the RF model (green line) aligned most closely with the ideal diagonal line. Quantitatively, the RF model achieved the lowest Brier score of 0.101 (SD = 0.011), indicating the minimal mean squared error in probability predictions compared to XGB (0.104) and LR (0.153).

Finally, Decision Curve Analysis (DCA) was employed to estimate the clinical utility of the models (**Figure 3F**). The DCA showed that the RF model provided the highest net benefit across a wide range of threshold probabilities compared to the other models and the “treat-all” or “treat-none” strategies. This suggests that using the RF model for decision-making would result in the best clinical outcomes.

In conclusion, considering discrimination, precision, calibration, and clinical utility, the RF model exhibited the best comprehensive capabilities and was selected as the final predictive model for this study.

### 3.4 RF model evaluation and validation

To rigorously assess the generalization ability and robustness of the selected RF model, we performed a multi-dimensional evaluation using training, validation, and independent test sets (refer to **Supplementary Tables S3**).

**Figure 4:**
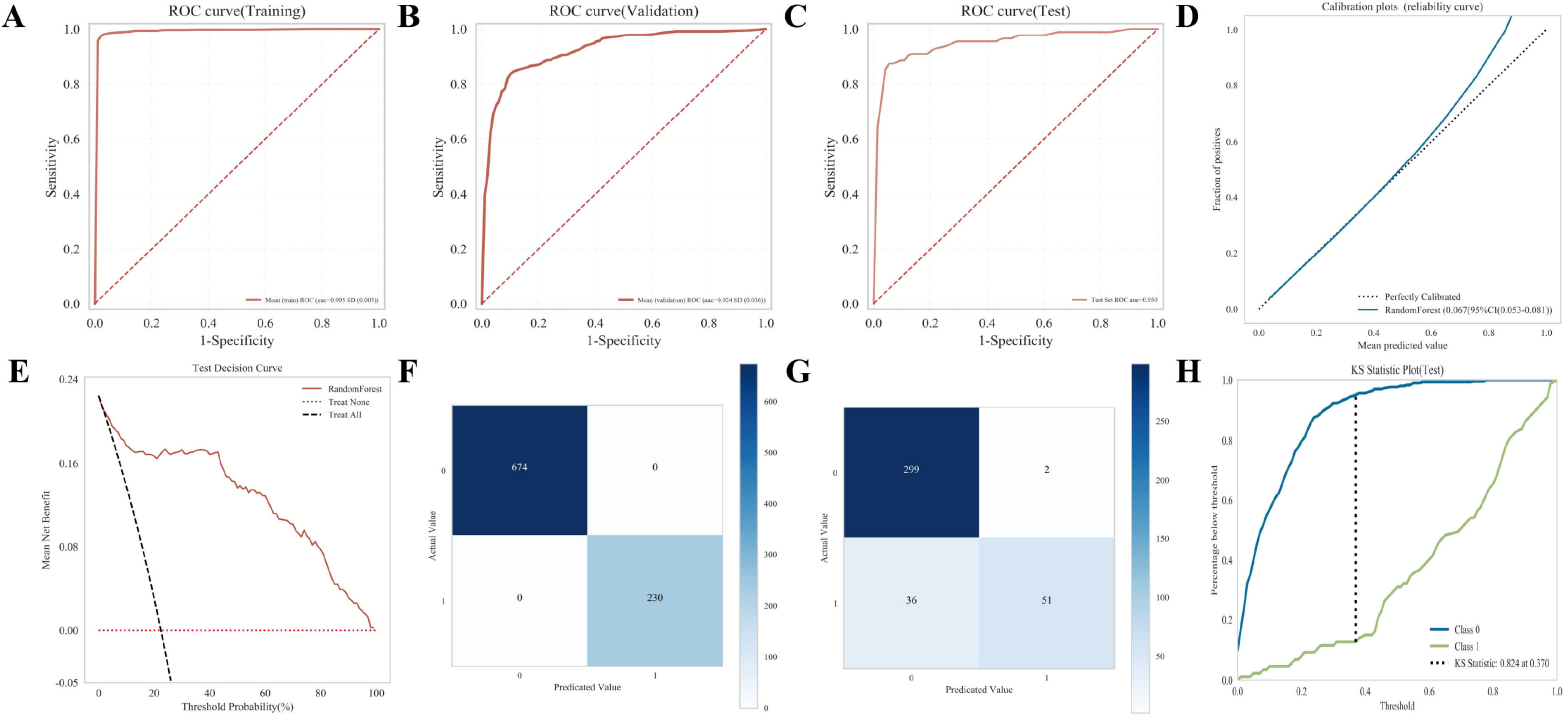
Assessment of model performance, calibration, and clinical utility. **(A–C)** ROC curves for the **(A)** training, **(B)** validation, and **(C)** independent test cohorts. The Area Under the Curve (AUC) is displayed in the bottom right of each panel. **(D)** Calibration curve showing the relationship between predicted probabilities and observed frequencies. The dotted line represents perfect calibration. **(E)** Decision Curve Analysis (DCA) for the test set, illustrating the clinical net benefit of the model. **(F, G)** Confusion matrices summarizing the prediction results (True/False Positives and Negatives) for the **(F)** training and **(G)** test datasets. **(H)** KS statistic plot demonstrating the maximum separation between the two classes distributions (Class 0 and Class 1).

First, the discrimination performance was evaluated using ROC curves across the different datasets. As shown in **Figure 4A**, the RF model achieved a near-perfect performance in the training set with a mean AUC of 0.995 (SD = 0.003). Crucially, this high level of discrimination was well-maintained in the validation set (**Figure 4B**, AUC = 0.924, SD = 0.036) and the independent test set (**Figure 4C**, AUC = 0.950). The consistency of high AUC scores across these datasets suggests that the model effectively learned the underlying patterns without suffering from significant overfitting.

The calibration of the model was further examined to ensure the reliability of the predicted probabilities. The calibration plot (**Figure 4D**) demonstrated excellent agreement between the predicted probabilities and the observed outcome frequencies, with the curve closely following the ideal 45-degree diagonal. The model achieved a low Brier score of 0.067 (95% CI: 0.053–0.081), indicating high accuracy in probability estimation.

To provide a detailed view of classification accuracy, confusion matrices were generated. **Figure 4F** displays the model’s performance on the training data, showing perfect classification with 674 true negatives and 230 true positives. In the test set (**Figure 4G**), the model continued to perform well, correctly identifying 299 true negatives and 51 true positives, with minimal false positives (n=2) and false negatives (n=36).

Furthermore, the Kolmogorov-Smirnov (KS) statistic was calculated to evaluate the model’s ability to separate positive and negative samples. As illustrated in **Figure 4H**, the RF model yielded a high KS statistic of 0.824 at a threshold of 0.370, confirming a significant distinction between the two classes distributions.

Finally, the clinical utility of the model was validated using DCA on the test set (**Figure 4E**). The decision curve showed that the RF model (red line) provided a higher net benefit than the “treat-all” or “treat-none” strategies across a wide range of threshold probabilities, underscoring its potential value in clinical decision-making.

### 3.5 Model interpretability and SHAP analysis

To overcome the “black box” nature of ML algorithms and provide clinical transparency, we employed SHAP analysis to elucidate the contribution of each feature to the RF model’s predictions.

**Figure 5:**
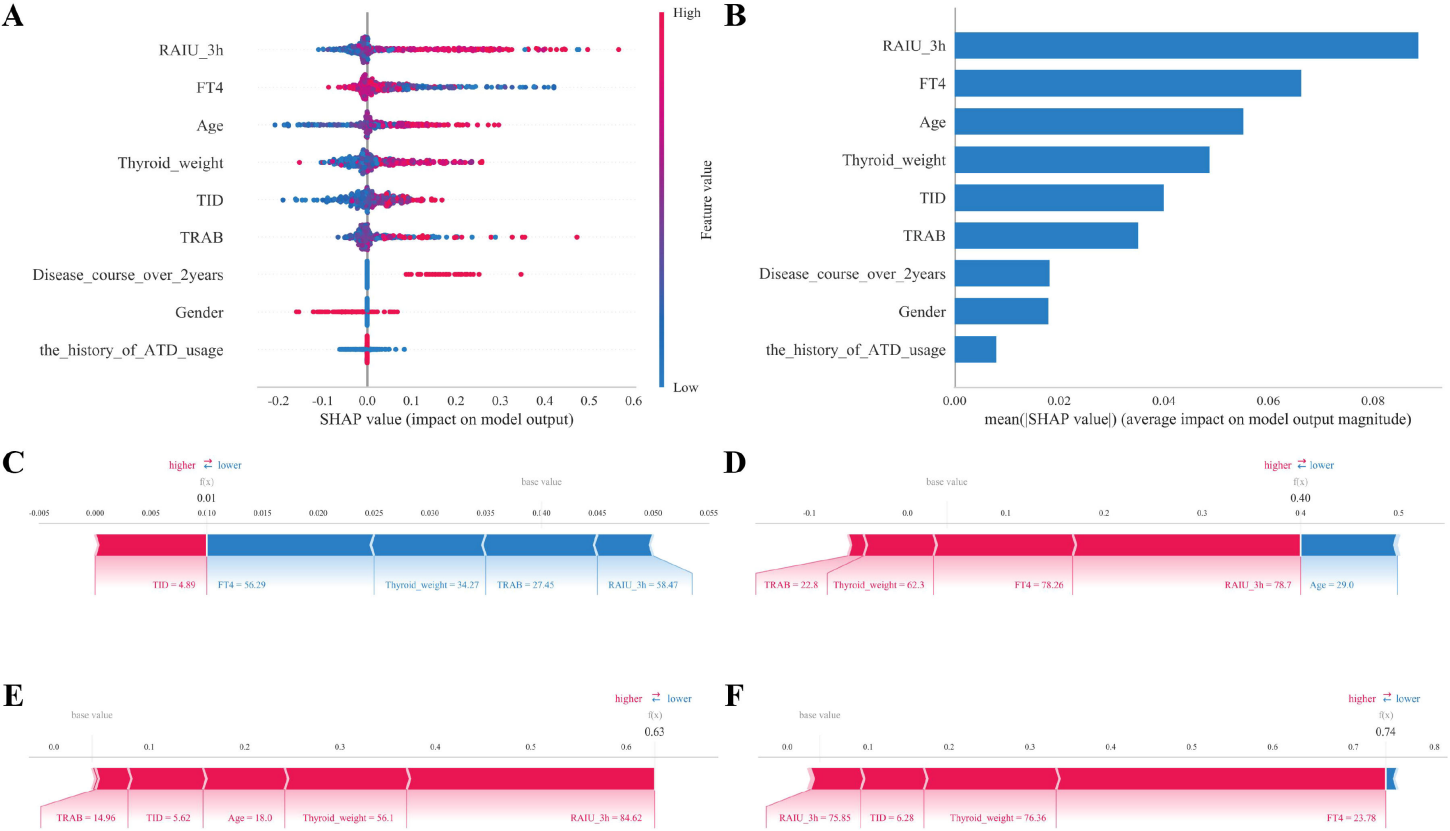
SHAP analysis for model explanation. **(A)** SHAP summary plot showing the impact of each feature on the model output. Points are colored by feature value (red for high, blue for low). **(B)** Bar plot of global feature importance ranked by the mean absolute SHAP value. **(C–F)** Individual force plots for local interpretability. The base value represents the average model output, and f(x) represents the predicted probability for the specific patient. Features in red contribute positively to the prediction, while features in blue contribute negatively. Cases shown include **(C)** low probability, **(D, E)** high probability, and **(F)** moderate probability.

The global importance of features is illustrated in the bar plot (**Figure 5B**), which ranks variables based on the mean absolute SHAP values. RAIU 3h was identified as the most influential predictor, followed by FT4, age, and thyroid weight. Other variables, such as TID, TRAB, disease course over 2 years, gender, and the history of ATD usage, showed relatively lower but non-negligible contributions to the model output.

The SHAP summary plot (**Figure 5A**) further visualizes the directionality and distribution of feature impacts. Each dot represents a sample, with color indicating the feature value (red for high, blue for low). For the top predictor, RAIU 3h, higher values (red dots) were predominantly associated with positive SHAP values, indicating a positive correlation with the predicted probability of the outcome. Similarly, higher age and thyroid weight generally contributed to an increased risk prediction. Conversely, for FT4, higher values (red dots) were clustered on the negative side of the SHAP axis, suggesting that elevated FT4 levels tend to lower the predicted probability, whereas lower FT4 levels contribute to a higher risk score.

To demonstrate how the model arrives at decisions for individual patients, SHAP force plots were generated for representative cases (**Figures 5C–F**). In these plots, red arrows indicate features that increase the prediction value, while blue arrows indicate features that decrease it.

**Figure 5C** shows a patient with a very low predicted probability (f(x)=0.01). The low risk score was primarily driven by the combined negative contributions (blue arrows) of RAIU 3h (58.47%), FT4 (56.29 pmol/L), and thyroid weight (34.27 g).

In contrast, **Figure 5D** illustrates a high-risk case (f(x)=0.74). Here, elevated RAIU 3h (75.85%) and thyroid weight (76.36 g) acted as strong positive drivers (red arrows), pushing the prediction towards a higher probability.

**Figure 5E** presents another high-risk patient (f(x)=0.63), where RAIU 3h (84.62%) again played a dominant role in increasing the model output. **Figure 5F** depicts a case with a moderate probability (f(x)=0.40). This prediction resulted from a balance between conflicting factors: while FT4 (78.26 pmol/L) and thyroid weight (62.3 g) pushed the score higher (red), RAIU 3h (78.7%) and age (29.0) exerted a downward influence (blue). These visualizations confirm that the RF model relies on clinically relevant markers, primarily RAIU 3h and FT4, to stratify patients effectively.

## 4 Discussion

This study developed and validated a RF model to predict the efficacy of initial RAI therapy in patients with GH, utilizing a comprehensive set of clinical, laboratory, and imaging parameters. The RF model demonstrated superior performance in discrimination, calibration, and clinical utility compared to other prominent ML algorithms, achieving an impressive AUC of 0.950 on an independent test set. Crucially, the application of SHAP analysis provided critical insights into the model’s decision-making process, highlighting the most influential features and their directional impact on individual predictions, thereby addressing the “black box” challenge inherent in complex ML models (20, 21).

RAI remains a cornerstone in the management of GH due to its high cure rates, non-invasive nature, and cost-effectiveness (22). However, determining the optimal RAI dose has historically been a significant challenge, with traditional fixed-dose regimens often failing to account for individual patient variability, leading to suboptimal outcomes or side effects (23). Our study reinforces the necessity of personalized dosing by identifying key patient-specific factors influencing treatment success. The achieved high predictive accuracy of the RF model underscores the potential of ML to overcome these traditional limitations by integrating multifactorial clinical data to inform tailored treatment strategies. This approach aligns with the growing emphasis on precision medicine in nuclear medicine, where diagnosis and treatment are increasingly molecularly targeted and individualized (24).

Our study identified RAIU 3h as the most influential predictor, with higher values generally associated with an increased likelihood of the predicted outcome (remission/non-remission, depending on the context of the model output). This is highly consistent with previous research, which recognizes RAIU, especially early uptake values, as a crucial indicator of the thyroid gland’s iodine-concentrating ability and radiosensitivity (25, 26). Studies have shown that low 24-hour RAIU implies a high cure rate, whereas high 24-hour RAIU indicates a high failure rate (26). Similarly, a high percentage uptake at 24 hours after a test dose of ^131^I administration has been identified as an influential predictor (25). A significantly elevated early uptake (3h) often indicates rapid iodine turnover, suggesting a hyper-functioning gland where the retention time of radioiodine may be insufficient to deliver the therapeutic absorbed dose. This aligns with the kinetic theory that rapid turnover reduces the effective half-life of I-131, thereby increasing the risk of treatment failure (Non-Remission).

The study confirmed thyroid weight as a significant positive predictor, indicating that larger thyroid glands are associated with a higher predicted probability of the outcome (potentially non-remission given the context of factors influencing treatment failure). This corroborates extensive literature demonstrating that larger thyroid gland size or weight is a well-established negative predictor for successful RAI therapy (27–29). Larger glands often correlate with increased radioresistance and higher rates of treatment failure due to the difficulty in delivering a sufficient and uniform radiation dose throughout the tissue. Our SHAP analysis specifically validated thyroid weight as a significant contributor, with higher values generally increasing the risk prediction. In previous research, the incidence of hypothyroidism in patients with non-palpable goiter was higher than in those with medium or large goiter, further supporting the influence of thyroid size (30).

Our model identified FT4 as a negative predictor, meaning higher FT4 levels were associated with a lower predicted probability of successful outcome. The SHAP summary plot visually reinforced this by showing that higher FT4 values contributed negatively to the predicted probability. This aligns with previous studies indicating that higher FT4 concentrations at presentation may correlate with a poorer response to RAI therapy or a higher likelihood of treatment failure. For instance, a study noted that successfully treated GD patients had a lower FT4 at presentation (25). Additionally, a negative association has been found between FT4 levels and disease remission after therapy discontinuation. However, some studies have noted no significant correlation between plasma P-Selectin levels and serum FT4 levels, suggesting that the direct interpretation of FT4’s role can be complex and may involve interactions with other factors (31).

Elevated TRAb levels were positively associated with the outcome in our multivariate analysis, suggesting that higher TRAb titers might predict a lower success rate or higher non-remission risk. This is highly consistent with earlier research, which has consistently linked high TRAb titers to reduced RAI efficacy and an increased risk of treatment failure (29). High TRAb levels suggest ongoing autoimmune activity that may counteract the therapeutic effect of RAI. A study found that the mean TRAb index of the hyperthyroid group was significantly higher than that of the euthyroid group, and TRAb index had a significant effect on the rate of hyperthyroidism after 3 months or later (32). Another study showed that the level of TRAb in the non-remission group was higher than that in the remission group. This study confirms TRAb as a critical prognostic marker, quantitatively integrated into the ML framework.

Our multivariate analysis found gender (male) to be a negative predictor, suggesting male patients may have a higher risk of non-remission compared to females. While some studies suggest no significant difference in cure rates between females and males, or efficacy not dependent on gender, others indicate varied associations (30, 33). Conversely, another study found that male gender was associated with treatment failure and was a main risk factor for early hypothyroidism (28, 29). These varied associations highlight the need for further investigation into gender-specific biological or clinical factors influencing RAI outcomes, with this study contributing to that nuanced understanding.

Age was found to be a positive predictor, implying that older age was associated with a higher predicted probability of the outcome (potentially remission). This finding is consistent with some prior research indicating that older patients may respond more favorably to RAI therapy (34). Successfully treated GD patients were younger than unsuccessfully treated ones in one study, while another found age to be a relative factor influencing RAI treatment efficacy, with older patients less likely to achieve clinical improvement (35). The effect of patient age as an influential predictor has been reported in other studies as well.

TID was positively associated with the outcome, which is broadly supported by studies demonstrating that higher RAI doses can increase success rates and achieve earlier treatment success. This aligns with the increasing clinical consensus that individualized dosing strategies, rather than fixed-dose regimens, lead to better patient outcomes (36, 37). The present study’s formula-based calculation method for RAI dose determination aims to personalize treatment by considering factors like thyroid size and iodine uptake, moving beyond the limitations of empirical fixed-dose approaches. This personalized approach is crucial because traditional fixed-dose methods often overlook individual patient differences, potentially leading to suboptimal outcomes or side effects.

The comprehensive comparison of six ML algorithms demonstrated the superior overall performance of the RF model. With an AUC of 0.908 in the validation cohort and 0.950 in the independent test set, along with excellent Average Precision, a low Brier score, and significant net benefit in DCA, the RF model exhibited robust discrimination and calibration capabilities. This aligns with previous research highlighting RF’s advantages in medical prediction due to its ability to handle high-dimensional data, complex interactions, and robustness against overfitting (38–40). Specifically in thyroid disease prediction, RF has consistently shown high accuracy and stable performance compared to other algorithms (41–43). A significant contribution of this study is the integration of SHAP analysis to provide interpretability for the RF model. The SHAP summary and dependence plots globally revealed the relative importance and directional impact of each feature, confirming that the model leverages clinically meaningful variables such as RAIU 3h, FT4, age, and thyroid weight. Furthermore, individual SHAP force plots illustrated how these features cumulatively drive predictions for specific patients, offering a transparent view of the model’s reasoning. This interpretability is crucial for fostering clinician trust and facilitating the clinical translation of AI-driven tools, as it allows healthcare professionals to understand why a particular prediction is made, rather than just what the prediction is (20, 21).

The developed interpretable RF model holds significant clinical utility for personalizing RAI therapy in GH patients. By accurately predicting the efficacy of initial RAI treatment, clinicians can make more informed decisions regarding patient selection, dose adjustment, and follow-up strategies. For instance, patients identified as high-risk for treatment failure by the model, perhaps due to large goiter size and high TRAb titers, could be considered for higher initial RAI doses or alternative treatments, potentially reducing the need for repeat RAI administrations or extended ATD use (29, 44, 45). Conversely, patients predicted to respond well might receive optimized lower doses, reducing radiation exposure while maintaining efficacy. This personalized approach aligns with the goal of precision medicine, enhancing therapeutic success, and minimizing side effects such as post-RAI hypothyroidism or the exacerbation of Graves’ orbitopathy (46). Furthermore, by reducing treatment failures and subsequent interventions, our model can contribute to resource optimization within healthcare systems, particularly in nuclear medicine departments. Improved efficacy predictions can lead to more efficient scheduling, reduced drug waste, shorter hospital stays, and better allocation of protective ward resources, which is especially pertinent in regions facing such constraints (47).

Despite the promising results, this study has several limitations. First, the exclusion of patients with massive goiters (>80g) limits the model’s generalizability to this specific subgroup. Second, as a single-center retrospective study, it carries inherent risks of selection bias and may limit the generalizability of the findings to diverse patient populations and healthcare settings. Although an independent test set was used for validation, external validation with multicenter, prospective cohorts is essential to confirm the robustness and applicability of the model across different demographics and clinical practices. Third, while a comprehensive set of clinical features was included, other potential confounders not collected in our dataset might influence RAI outcomes. Future studies could explore the integration of additional multimodal data, such as genomic markers, advanced imaging features (radiomics beyond thyroid weight), or dynamic physiological data, using advanced data fusion techniques to build even more sophisticated and accurate predictive models. Fourth, our outcome classification into “Remission” versus “Non-Remission” is a binary simplification of a more complex clinical spectrum, which includes distinct states like euthyroidism and hypothyroidism. Future research could investigate multi-class classification models to predict these specific outcomes more granularly, offering more nuanced guidance for post-treatment management. Finally, future efforts should focus on transitioning these predictive models into real-time clinical decision support systems. These systems could dynamically update predictions based on evolving patient data, provide actionable recommendations at the point of care, and facilitate shared decision-making between clinicians and patients. This transition would also necessitate rigorous evaluation of the ethical implications, data security, and patient privacy within such AI-driven healthcare applications.

In conclusion, our study successfully developed an interpretable RF model that accurately predicts the efficacy of initial RAI therapy in GH patients. By identifying key predictive factors and providing transparent explanations through SHAP analysis, this model represents a significant step towards personalized medicine, promising improved clinical outcomes, enhanced resource utilization, and a more scientific approach to managing GH.

## Data Availability

The original contributions presented in the study are included in the article/**Supplementary Material**. Further inquiries can be directed to the corresponding authors.
