# Supplementary material for: From data to decision: an interpretable machine learning model for optimizing RAI therapy in Graves’ hyperthyroidism
Source: Front Endocrinol (Lausanne). 2026 Jan 26;16:1711029. doi: 10.3389/fendo.2025.1711029 (PMC12883413; doi:10.3389/fendo.2025.1711029)
Supplement: Supplementary file 1 [file DataSheet1.doc]

| Model | AUC(SD) | cutoff(SD) | Accuracy(SD) | Sensitivity(SD) | Specificity(SD) | PPV(SD) | NPV(SD) | F1 score(SD) | Kappa(SD) | Group |
| --- | --- | --- | --- | --- | --- | --- | --- | --- | --- | --- |
| XGB | 0.986(0.002) | 0.394(0.026) | 0.969(0.002) | 0.925(0.009) | 0.984(0.005) | 0.952(0.014) | 0.975(0.003) | 0.938(0.004) | 0.918(0.005) | train |
| XGB | 0.892(0.031) | 0.394(0.026) | 0.854(0.018) | 0.661(0.017) | 0.920(0.022) | 0.741(0.056) | 0.888(0.006) | 0.698(0.031) | 0.602(0.044) | valiation |
| LR | 0.789(0.011) | 0.237(0.014) | 0.708(0.010) | 0.759(0.027) | 0.691(0.021) | 0.456(0.010) | 0.894(0.008) | 0.570(0.006) | 0.369(0.010) | train |
| LR | 0.774(0.057) | 0.237(0.014) | 0.688(0.055) | 0.704(0.072) | 0.682(0.055) | 0.435(0.062) | 0.870(0.035) | 0.537(0.065) | 0.322(0.102) | valiation |
| LGBM | 0.670(0.008) | 0.256(0.000) | 0.705(0.006) | 0.597(0.021) | 0.743(0.010) | 0.442(0.008) | 0.844(0.006) | 0.508(0.010) | 0.304(0.013) | train |
| LGBM | 0.667(0.034) | 0.256(0.000) | 0.704(0.027) | 0.591(0.064) | 0.742(0.032) | 0.439(0.038) | 0.842(0.020) | 0.503(0.044) | 0.299(0.060) | valiation |
| RF | 0.993(0.002) | 0.582(0.012) | 0.969(0.006) | 0.891(0.018) | 0.996(0.004) | 0.986(0.014) | 0.964(0.006) | 0.936(0.012) | 0.916(0.016) | train |
| RF | 0.908(0.039) | 0.582(0.012) | 0.854(0.020) | 0.478(0.079) | 0.982(0.004) | 0.900(0.022) | 0.847(0.019) | 0.621(0.071) | 0.542(0.076) | valiation |
| AdaBoost | 0.898(0.011) | 0.495(0.002) | 0.829(0.014) | 0.816(0.071) | 0.834(0.039) | 0.632(0.040) | 0.931(0.021) | 0.708(0.016) | 0.591(0.022) | train |
| AdaBoost | 0.777(0.059) | 0.495(0.002) | 0.741(0.040) | 0.617(0.065) | 0.783(0.064) | 0.504(0.065) | 0.858(0.018) | 0.550(0.039) | 0.373(0.065) | valiation |
| DT | 0.948(0.008) | 1.000(0.000) | 0.960(0.005) | 0.922(0.018) | 0.974(0.007) | 0.923(0.018) | 0.973(0.006) | 0.922(0.009) | 0.896(0.012) | train |
| DT | 0.755(0.018) | 1.000(0.000) | 0.822(0.008) | 0.617(0.053) | 0.892(0.021) | 0.663(0.029) | 0.873(0.013) | 0.637(0.023) | 0.520(0.023) | valiation |

Table S1. Summary of multi-model classification results

Table S2. Delong test P-value mean table

| Model | XGB | LR | LGBM | RF | AdaBoost | DT |
| --- | --- | --- | --- | --- | --- | --- |
| XGB | NA | 1.0 | 1.0 | 1.0 | 1.0 | 1.0 |
| LR | 1.0 | NA | 1.0 | 1.0 | 1.0 | 1.0 |
| LGBM | 1.0 | 1.0 | NA | 1.0 | 1.0 | 1.0 |
| RF | 1.0 | 1.0 | 1.0 | NA | 1.0 | 1.0 |
| AdaBoost | 1.0 | 1.0 | 1.0 | 1.0 | NA | 1.0 |
| DT | 1.0 | 1.0 | 1.0 | 1.0 | 1.0 | NA |

Table S3. Summary of RF classification results

| AUC(SD) | cutoff(SD) | Accuracy(SD) | Sensitivity(SD) | Specificity(SD) | PPV(SD) | NPV(SD) | F1 score(SD) | Kappa(SD) | Group |
| --- | --- | --- | --- | --- | --- | --- | --- | --- | --- |
| 0.995 (0.003) | 0.572 (0.025) | 0.974 (0.003) | 0.907 (0.017) | 0.997 (0.002) | 0.992 (0.006) | 0.969 (0.006) | 0.947 (0.008) | 0.930 (0.010) | train |
| 0.924 (0.036) | 0.572 (0.025) | 0.860 (0.021) | 0.496 (0.064) | 0.984 (0.011) | 0.911 (0.061) | 0.851 (0.017) | 0.640 (0.064) | 0.563 (0.073) | valiation |
| 0.95 | 0.61 | 0.907 | 0.609 | 0.993 | 0.964 | 0.898 | 0.746 | 0.693 | test |

**The parameter selection for each model is as follows:**

XGBClassifier: AUC=0.9834650856389986; Model parameters:

colsample_bytree (feature subsample rate): 1

learning_rate (learning rate): 0.3

max_depth (maximum tree depth): 8

min_child_weight (minimum child weight sum): 2

n_estimators (number of base learners): 20

reg_lambda (L2 regularization coefficient): 0.5

subsample (subsample rate): 1

LogisticRegression: AUC=0.7882850241545892; Model parameters:

C (regularization factor): 0.09999999999999999

l1_ratio (ElasticNet mixing parameter): None

max_iter (number of iterations): 50

penalty (regularization type): l2

solver (solver): lbfgs

tol (convergence metric): 0.0001

LGBMClassifier: AUC=0.6696441679211687; Model parameters:

boosting_type (algorithm type): gbdt

learning_rate (learning rate): 0.001

max_depth (maximum tree depth): 1

n_estimators (maximum number of trees): 5

num_leaves (maximum number of leaves): 5

RandomForestClassifier: AUC=0.9891703387390522; Model parameters:

criterion (metric): gini

max_depth (maximum tree depth): None

max_features (maximum number of features): sqrt

min_impurity_decrease (minimum impurity decrease): 0.0

min_samples_leaf (minimum samples at leaf nodes): 1

min_samples_split (minimum samples required to split an internal node): 2

n_estimators (number of trees): 100

AdaBoostClassifier: AUC=0.8971844950012494; Model parameters:

learning_rate (learning rate): 1.0

n_estimators (number of base models): 50

DecisionTreeClassifier: AUC=0.9474014786993769; Model parameters:

criterion (splitting function): log_loss

max_depth (maximum tree depth): 20

min_samples_leaf (minimum samples at leaf nodes): 1

min_samples_split (minimum samples required to split): 2
